# Automated peripheral nerve segmentation for MR-neurography

**DOI:** 10.1186/s41747-024-00503-8

**Published:** 2024-08-26

**Authors:** Nedim Christoph Beste, Johann Jende, Moritz Kronlage, Felix Kurz, Sabine Heiland, Martin Bendszus, Hagen Meredig

**Affiliations:** 1 Institute of Neuroradiology, University Hospital of Heidelberg, Heidelberg, Germany; 2 DKFZ German Cancer Research Center, Heidelberg, Germany

**Keywords:** Artificial intelligence, Magnetic resonance imaging, Neural networks (computer), Peripheral nervous system, Sciatic nerve

## Abstract

**Background:**

Magnetic resonance neurography (MRN) is increasingly used as a diagnostic tool for peripheral neuropathies. Quantitative measures enhance MRN interpretation but require nerve segmentation which is time-consuming and error-prone and has not become clinical routine. In this study, we applied neural networks for the automated segmentation of peripheral nerves.

**Methods:**

A neural segmentation network was trained to segment the sciatic nerve and its proximal branches on the MRN scans of the right and left upper leg of 35 healthy individuals, resulting in 70 training examples, via 5-fold cross-validation (CV). The model performance was evaluated on an independent test set of one-sided MRN scans of 60 healthy individuals.

**Results:**

Mean Dice similarity coefficient (DSC) in CV was 0.892 (95% confidence interval [CI]: 0.888–0.897) with a mean Jaccard index (JI) of 0.806 (95% CI: 0.799–0.814) and mean Hausdorff distance (HD) of 2.146 (95% CI: 2.184–2.208). For the independent test set, DSC and JI were lower while HD was higher, with a mean DSC of 0.789 (95% CI: 0.760–0.815), mean JI of 0.672 (95% CI: 0.642–0.699), and mean HD of 2.118 (95% CI: 2.047–2.190).

**Conclusion:**

The deep learning-based segmentation model showed a good performance for the task of nerve segmentation. Future work will focus on extending training data and including individuals with peripheral neuropathies in training to enable advanced peripheral nerve disease characterization.

**Relevance statement:**

The results will serve as a baseline to build upon while developing an automated quantitative MRN feature analysis framework for application in routine reading of MRN examinations.

**Key Points:**

- Quantitative measures enhance MRN interpretation, requiring complex and challenging nerve segmentation.
- We present a deep learning-based segmentation model with good performance.
- Our results may serve as a baseline for clinical automated quantitative MRN segmentation.

**Graphical Abstract:**

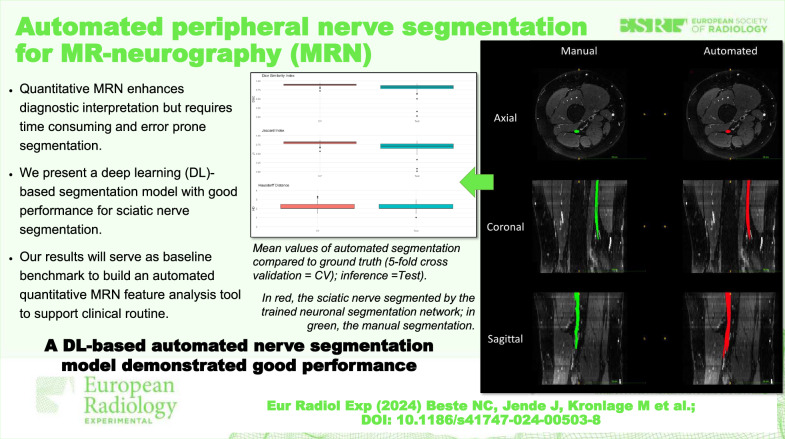

## Background

Magnetic resonance neurography (MRN) is increasingly used as an additional diagnostic tool for peripheral nerve examination, complementing electrodiagnostic studies and clinical evaluation [1–9]. Especially in deep regions that are hard to assess by electrodiagnostic studies (*e.g*., lumbosacral plexus) [10, 11] or in inconclusive clinical examinations, MRN studies can improve the diagnosis of neuropathies. Hereby, the usage of specific MR imaging-based measures such as mean signal intensity or parameters derived from diffusion-weighted imaging of peripheral nerves [12–15] can enhance MRN interpretation and improve diagnostic accuracy. However, manual segmentation as a prerequisite for this quantitative image analysis is a labor-intensive task to be added to an already complex process of image acquisition and interpretation. Thus, the applicability of quantitative MRN in clinical practice is still limited.

A small number of studies have focused on the semiautomatic analysis of different imaging features in MRN [16–18], but they still rely on the manual selection of a particular region of interest. This renders these methods prone to decreased reproducibility, especially if measurements are not conducted at the exact same location by different readers. An alternative approach is the complete segmentation of the peripheral nerve of interest. Here, the whole examination volume is considered, which offers further insights by granting access to global imaging features and offering more possibilities for automated analysis.

With recent advances in machine learning and artificial intelligence, reliable fully automated segmentation of multiple organs of the human body across different imaging modalities now becomes feasible by the application of neural network-based deep learning algorithms. The U-Net architecture [19] has been the first breakthrough demonstrating excellent results in biomedical image segmentation of electron microscopy stacks. Since then, multiple modified or alternative neural network-based approaches have been proposed with the standard U-Net architecture still being among the most successful [20]. With a fully automatic segmentation and analysis, reader disagreement-related issues are effectively mitigated, and supportive application in routine settings becomes feasible. The value of this artificial intelligence-assisted approach in clinically relevant decision processes has already been demonstrated multiple times with applications in many different disciplines [21–27].

Balsiger et al [1] were the first to apply such a neural network-based approach to peripheral nerve segmentation with good results. Their study included a cohort of 42 patients and 10 healthy individuals. They applied a custom fully convolutional network and evaluated the performance in a 4-fold cross-validation. While showing promising results, the study was conducted on a relatively small and heterogenous cohort and the evaluation lacked an independent test set.

The current study builds upon these results by developing a peripheral nerve segmentation tool for MRN using a fully automated neural network to establish a baseline performance evaluation on healthy individuals. To the best of our knowledge, the largest cohort of individuals so far was included for training, and this is the first study to validate its results on an independent test set.

## Methods

Approval of the Heidelberg University local Ethics Board and patient informed consent, including the reference to human and animal rights declarations and regulations were obtained for all subjects of this study.

### Participants

Ninety-five healthy participants who had undergone MRN at the Department of Neuroradiology at Heidelberg University were retrospectively included in this study. The training data set included MR scans from 35 healthy participants, aged 32 ± 6 years (mean ± standard deviation), with a males/females ratio of 1:0.8; the test data set included 60 healthy participants, aged 50 ± 17 years, with a males/females ratio of 1:1. Exclusion criteria were peripheral or central neurological diseases (neuropathies, stroke, disc herniation, and myelopathy) systemic diseases (inflammatory diseases, diabetes, and vasculitis) and exclusion criteria for MR examinations. All participants gave informed consent to participate in the study.

### Image acquisition

Training and test set underwent large coverage two-dimensional (2D) T2-weighted MRN of the upper leg (Magnetom TIM-TRIO 3-T scanner, Siemens Healthineers (Heidelberg, Germany)) with spectral fat saturation. An anterior knee coil and a built-in spine coil were used. Detailed sequence parameters can be found in Table 1. In the training set, 35 participants were examined on both sides while the 60 participants of the test set were examined on one randomly chosen side (31 right and 29 left). To summarize, the training set included 70 scans, and the test data set had 60 scans. Data acquisition and subject recruitment for the two cohorts were conducted in the context of previous distinct and independent studies and at differing points in time. Sequence parameters have been updated in the context of the different main investigation endpoints of the respective studies and are therefore distinct from each other in the training and test set. This ensured that the test dataset remained independent from the training dataset, as the parameters were tailored to the specific requirements of each investigation.

**Table 1:** Standard sequence parameters for training test and data set

Parameter	Training	Test
Repetition time (ms)	5,970	7,000
Echo time (ms)	55	55
Field of view (mm^2^)	160 × 160	160 × 160
Matrix size	512 × 512	512 × 333
Slice thickness (mm)	4	3.5
Interslice gap (mm)	0.35	0.35
Voxel size (mm^3^)	0.3 × 0.3 × 4.0	0.3 × 0.5 × 3.5 mm^3^
Number of slices	24	41

### Manual segmentation

Using ITK-SNAP version 3.8.0 (http://www.itksnap.org/pmwiki/pmwiki.php) the sciatic nerve including its proximal tibial and peroneal branches was manually segmented twice by the same reader (N.B.) within an interval of 34 weeks (about 8 months) in-between individual reads. The intra-rater correlation was assessed by computing the Dice similarity coefficient (DSC) [28, 29], Jaccard index (JI) [30], and Hausdorff distance (HD) [31] of the mean intensity across all segmented voxels.

### Neural network development

Preprocessing, training, and inference were conducted using the Python package nnU-Net (Version 1.0, [20]).

### Preprocessing

A 2D and a three-dimensional (3D)-nnU-Net configuration was trained. Input for preprocessing was the upper leg MR scans of the training set and the segmentation files in the Neuroimaging Informatics Technology Initiative Format (nifti 1). The MR scans of the test data set were preprocessed later during inference. Every examination of the training set contained 35 images resulting in a shape of 35 × 510 × 510 voxels. Test set scans had a shape of 41 × 510 × 510. Voxel spacing was 3.85 × 0.32 × 0.32 mm^3^ for test and training. All images were intensity normalized by subtraction of the mean and subsequent division by standard deviation. More details for training and test sets can be found in Table 2.

**Table 2:** Segmentation model differences between 2D and 3D training data set and test data set

Parameter	2D training model	3D training model	Test set
Target spacing (mm^3^)	3.85 × 0.32 × 0.32	3.85 × 0.32 × 0.32	3.85 × 0.32 × 0.32
Patch size	512 × 512	20 × 320 × 256	20 × 320 × 256
Batch size	12	2	2

*2D* Two-dimensional, *3D* Three-dimensional

### Training and inference

The training was executed in a 5-fold cross-validation scheme for a 2D- and a 3D-architecture: first, an ensemble of five individual models was trained in a 2D-architecture for 150 epochs until model conversion. Additionally, an ensemble of five models in a 3D-full-resolution architecture was trained. Due to the increased model size, it here took 500 epochs until model conversion. Accuracy and loss curves for each model can be found in Supplementary Fig. S1 and architectural details in Supplementary Table S1. Patch size was determined at 512 × 512 voxels for the 2D model with a batch size of 12 and 20 × 320 × 256 voxels for the 3D model with a batch size of 2.

The training was executed on a computer workstation equipped with two NVIDIA Quadro RTX 8000 with an Intel(R) Xeon(R) Gold 6258 R CPU @ 2.70 GHz and 380 GB RAM. The 2D training took ~1 day in total, 3D training ~3 days in total. The maximum VRAM required by the 2D model was 7.7 GB and 11.5 GB for the 3D model.

For training a compound loss function consisting of Dice loss and cross-entropy loss as described in detail in [20] was used, combined with a standard stochastic gradient descent optimizer with a Nesterov momentum [32] of µ −0.99 and an initial learning rate of 0.01 following a polynomial learning rate schedule.

For data augmentation, the following techniques were applied: scaling and rotation with randomly defined rotation angles between -30° and 30° for the 3D and from -180° to 180° for the 2D model and scaling factors between 0.7 and 1.4 and probability of 0.16 each. Gaussian noise was adopted adding a variance of randomly chosen factors between 0 and 0.1 to the voxel intensity of an individual voxel with a probability of 0.15. Gaussian blur was employed with a width of 0.5–1.5 voxel and a probability of 0.1 per sample. Brightness and contrast were augmented by multiplying the voxel intensities by a range of randomly chosen factors between 0.7 and 1.3, respectively 0.65 and 1.5, with a probability of 0.15. The low resolution was simulated with a probability of 0.1 per sample by downsampling using nearest neighbor interpolation by a range of randomly chosen factors between 1 and 2 and cubic interpolation for the following upsampling. In addition, Gamma augmentation was utilized augmenting the patch intensity by a range of randomly chosen factors between 0 and 1 and nonlinear transformation of voxel intensity with a randomly chosen factor between 0.7 and 1.5. Finally, mirroring was applied with a probability of 0.5 to all axes of the patch.

For inference, an ensembled patch-based prediction of all five models was utilized by employing a connected component analysis-based postprocessing scheme as described in the original nnU-Net Publication [20].

### Statistical analysis

Intra-rater variability for ground truth segmentation was measured and the similarity of both training and test images to the ground truth was analyzed by calculating DSC, JI, and HD [31, 33] of the mean intensity of the segmented voxels using Python (Jupyter Notebook version 6.4.5) using Python modules “nibabel” (version 4.0.1) and “numpy” (version 1.20.3).

The following analysis was conducted with R (version 4.2.1): DSC, JI, and HD of the test and training set as well as the labels were tested for normal distribution using the Shapiro–Wilk test (Package “stats”, version 4.2.1). Bootstrapping was utilized for non-normally distributed data. Differences of mean DSC, JI, and HD for the validation images, test images, and labels were computed with (bootstrapped-) two-sample *t*-tests (R package “MKinfer” Version 1.0). A *p*-value below 0.05 was considered statistically significant; 95% CIs were calculated using the “MKinfer” package version 1.0.

## Results

Across all models, DSC, JI, and HD were not normally distributed. Details can be found in Supplementary Table S2.

Our results revealed a high intra-rater correlation with a mean DSC across images of 0.968 (95% CI: 0.964–0.972), mean JI of 0.940 (95% CI: 0.931–0.947), and mean HD of 1.733 (95% CI: 1.646–1.822) (Table 3) was computed. Bootstrapped two-sided *t*-tests revealed differences between the 2D and 3D models of DSC (*p* = 0.005) and JI (*p* = 0.007) (Table 4). The mean DSC in cross-validation was 0.882 (95% CI: 0.877–0.888) for the 2D model and 0.892 (95% CI: 0.888–0.897) for the 3D model, with JI of 0.792 (95% CI: 0.783–0.800) for the 2D model and 0.806 (95% CI: 0.799–0.814) for the 3D model. In addition, the mean HD was 2.180 (95% CI: 2.121–2.230) and 2.146 (95% CI: 2.184–2.208), respectively.

**Table 3:** Intra-rater correlation described by Dice similarity index, JI, and HD (mean of all images)

Metric	Point value	95% CI
Dice similarity index	0.968	0.964–0.972
JI	0.940	0.931–0.947
HD	1.733	1.646–1.822

**Table 4:** Cross-validation results

Model	Metric	Point value	95% CI	*p*-value
2D	Dice similarity index	0.882	0.877–0.888	0.005
	JI	0.792	0.783–0.800	0.007
	HD	2.180	2.121–2.230	0.439
3D	Dice similarity index	0.892	0.888–0.897	0.005
	JI	0.806	0.799–0.814	0.007
	HD	2.146	2.084–2.208	0.439

Mean values of model prediction on training data set compared to training ground truth segmentation (two samples bootstrapped *t-*test to compare 2D and 3D model)

**Fig. 1:**
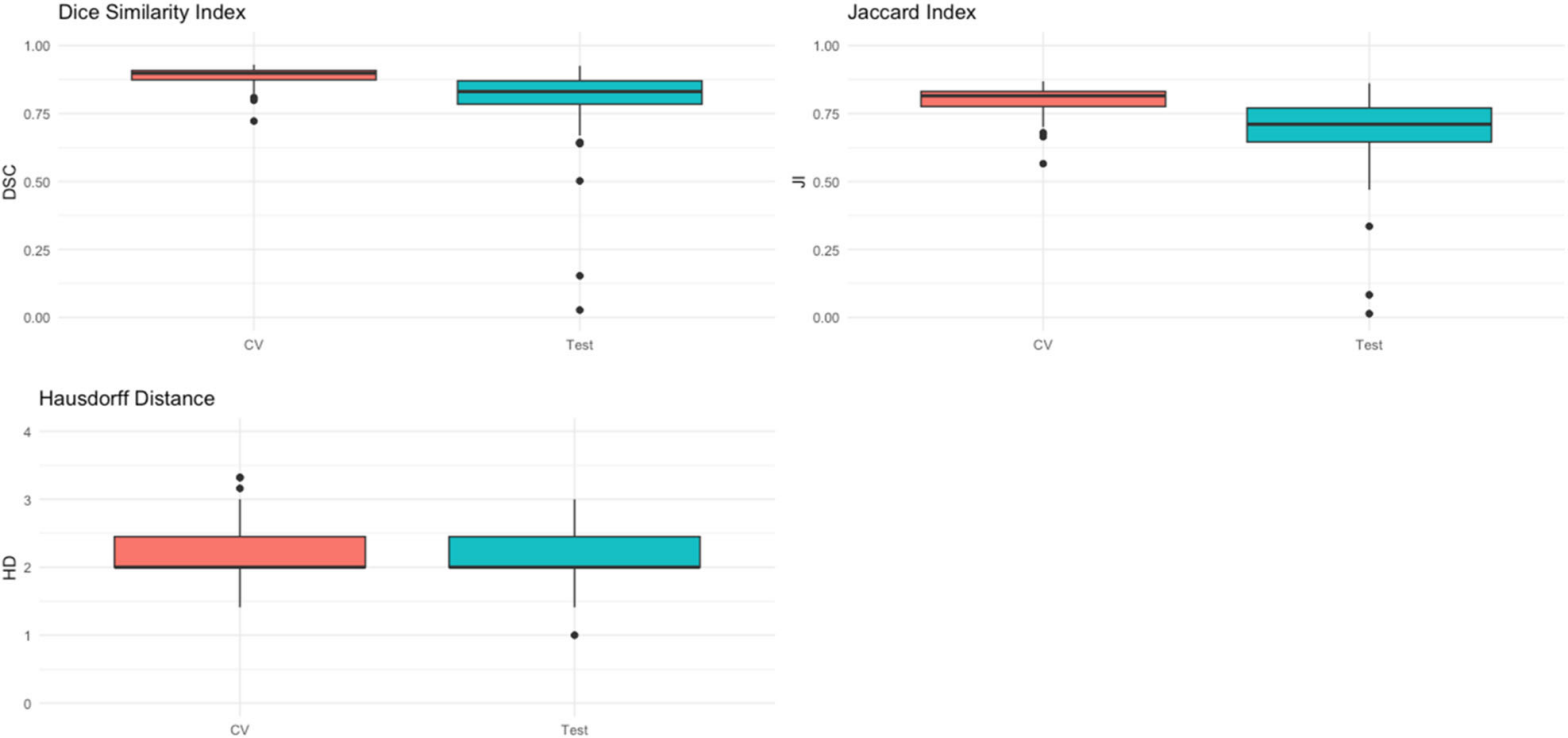
Dice similarity index, JI, and HD of 5-fold cross-validation and inference (test) when compared to ground truth segmentation

**Fig. 2:**
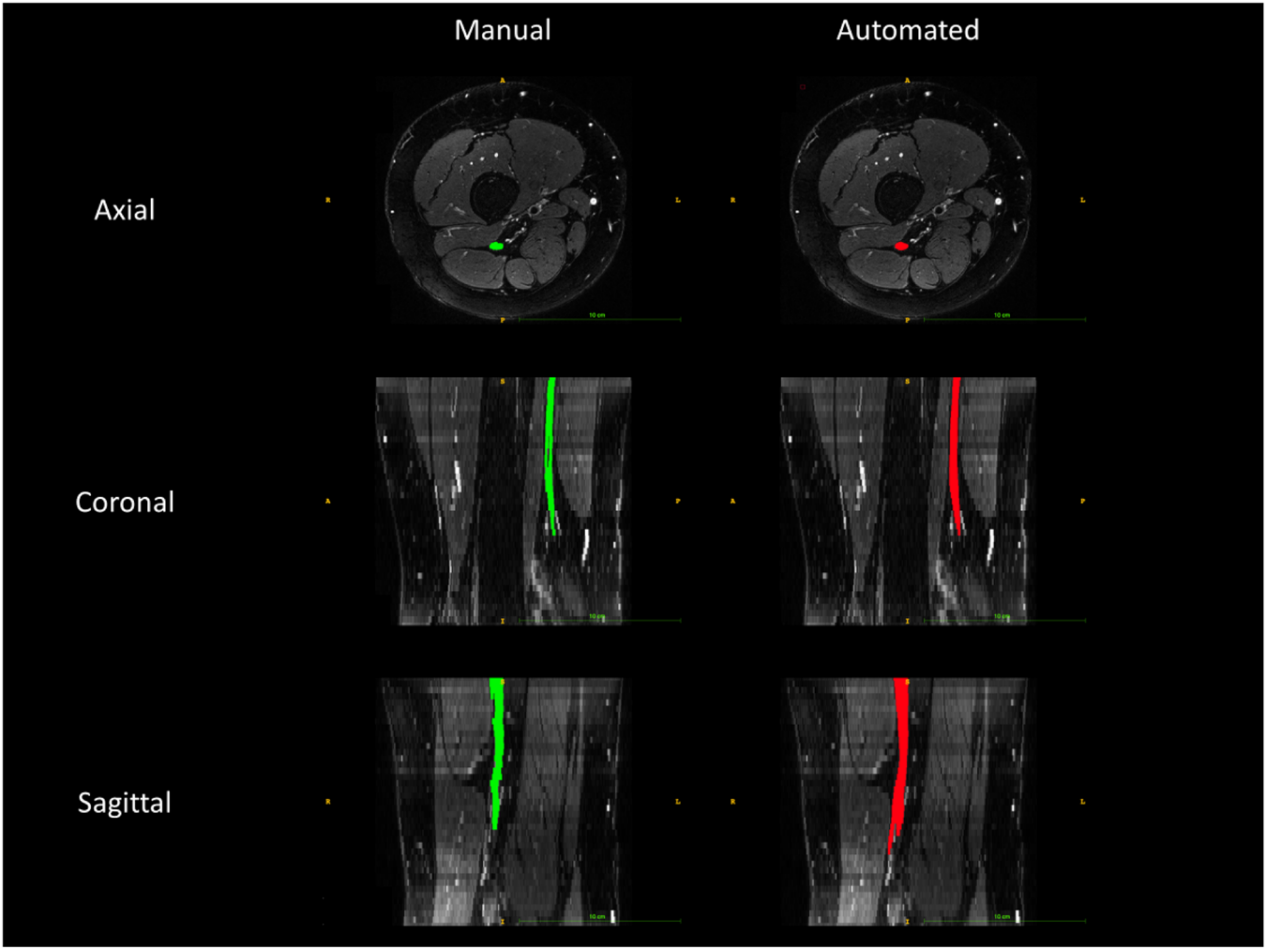
Automated *versus* manual nerve segmentation. The figure shows in red the sciatic nerve segmented by the trained neuronal segmentation network and in green the manual segmentation in the axial (upper row), coronal (middle row), and sagittal (bottom row) view

The 3D model was used for inference. The mean DSC on the test set was 0.789 (95% CI: 0.760–0.815) with a mean JI of 0.672 (95% CI: 0.642–0.699) and mean HD of 2.118 (95% CI: 2.047–2.190). Two sample bootstrapped *t*-tests showed lower DSC (*p* < 0.001) and JI (*p* < 0.001) on the test set compared to the training set (Table 5 and Figs. 1 and 2).

**Table 5:** Cross-validation vs test results

Model	Metric	Mean	95% CI	*p*-value
Cross-validation	Dice similarity index	0.888	0.884–0.891	< 0.001
	JI	0.799	0.794–0.805	< 0.001
	HD	2.163	2.120–2.206	0.307
Test	Dice similarity index	0.789	0.760–0.815	< 0.001
	JI	0.672	0.642–0.699	< 0.001
	HD	2.118	2.047–2.190	0.307

Mean values of model prediction for training data compared to training ground truth segmentation (cross-validation) *versus* mean values of model prediction on test data set compared to test ground truth segmentation (test results). Two-sample bootstrapped *t-*test was used to compare test and cross-validation

## Discussion

In this study, a U-net-based segmentation model for the task of sciatic nerve segmentation has been trained. A high DSC of 0.888 (with a JI of 0.799) was reached in cross-validation and a good DSC of 0.789 (with a JI of 0.672) was achieved on the independent test set. The HDs in cross-validation and on the test set results are high, reaching values of 2.163 and 2.118, respectively. Intra-rater correlation between two successive manual segmentations of the training and test data depicted a high DSC of 0.968 (with a JI of 0.940) and an HD of 1.733.

The high DSC in cross-validation is in line with the findings of earlier studies [1]. Moreover, the good DSC between ground truth and the prediction on the test set shows robust generalization capabilities of the model albeit the, in general, machine learning terms, limited amount of training data. The high intra-rater correlation between two successive manual segmentations of the training and test data depicts the high quality of training and evaluation labels. The fact that the 3D model variant outperformed the 2D variant was expected, since image information from adjacent layers is allowed to contribute to the identification of nerve pathways in the 3D architecture, despite the anisotropic acquisition technique of the fat-saturated 2D TSE sequences used in this study.

A robust segmentation model for sciatic nerve segmentation is a desirable premise for further advanced analysis methods in MRN. As previously shown, MRN interpretation can be improved by the usage of specific MR measures like the mean intensity of the nerve or parameters derived from diffusion-weighted imaging of peripheral nerves [12–15, 34, 35]. For the purpose of advanced imaging feature analysis in MRN, Felisaz et al [16, 17] presented a semiautomatic approach for analyzing imaging features in the specific case of patients with diabetic neuropathy. Rossi et al [18] distinguished healthy nerves from nerves of patients with carpal and cubital syndrome using radiomics-based intraneural feature analysis. Both examples are limited to a specific use case and the time-consuming task of the manual region of interest definition remains a disadvantage for clinical use.

As an alternative approach, automatic nerve segmentation is an investigator-independent method for advanced image analysis methods in clinical routine settings where manual or semiautomatic methods would complicate and prolong the diagnostic process. With recent advances in artificial intelligence, reliable fully automated segmentation of multiple organs of the human body across different imaging modalities has become feasible by the application of neural network-based deep learning algorithms. The main difficulty in the specific area of MRN, however, is the very limited sample size and small and curved structure of peripheral nerves. Compared to dataset sizes in classical machine learning and deep learning research, the medical field in general suffers from limited data availability due to data privacy regulations. In MRN, this issue is aggravated by the fact that the imaging technique is only used in specific clinical and diagnostic circumstances and only performed in specialized institutions where trained experts are available. While research in medical image segmentation in general has found widespread attention in recent years, the focus has been mostly on tasks where comparatively large datasets are available [36–38].

To the best of our knowledge, regarding the application of deep learning-based image segmentation on MRN images, there is in fact only one publication by Balsiger et al available [1]. In their pioneering work, they trained a customized fully convolutional network architecture on a dataset of 52 individuals in a 4-fold cross-validation scheme. While the results indicate good performance, evaluation on an independent test set of the model was not conducted, presumptively due to the already small amount of data available. With a training set size of 70 examples, our segmentation model has been trained on the—to our knowledge—to date largest cohort of individuals, and this study is the first to evaluate the performance of the model on an independent test set of 60 individuals. Opposed to Balsiger et al’s study, our model was currently developed only for the segmentation of the sciatic nerve of healthy individuals. Including pathologies in the initial training of neural segmentation networks constitutes a more challenging segmentation task for the network due to a wider variability in the appearance of nerve tissue in MRN regarding morphological, intensity, and contrast information. On the one hand, this is a crucial requirement to enable flexibility and generalization capabilities of the model to unseen cases. On the other hand, especially in the case of very limited data, large inhomogeneities in the training set may decrease overall performance. The concept of the current study was designed as a reference for depicting the baseline performance in the optimal case of relatively homogenous test and training sets supplying the subsequent development of more versatile models developed on more heterogenous datasets.

The main limitation of this study is the restriction to the examinations of healthy individuals. The current segmentation model might not provide robust results on the segmentation of pathologically altered peripheral nerves. Extending training and test data to patients with neuropathy will therefore be a focus of future work.

Another limitation is the constraint on the segmentation of nerve tissue alone. As signal intensities in MR imaging examinations may vary depending on scanner type, coils used, and local environmental factors, the reference for the evaluation of the nerve’s signal intensity in MRN is the intensity of adjacent muscle tissue [39]. To be of use in the process of further MRN analysis, a segmentation algorithm should be able to not only segment nerve tissue, but also identify regions of skeletal musculature for reference as well. However, as muscle tissue comprises a much larger fraction in the examination volume there are alternative possibilities for identifying areas of skeletal musculature, for example by simple clustering techniques of the signal intensities in an examination volume which do not require the labor-intensive process of manual generation of training labels for neural network-based segmentation model training.

A third limitation is that the training cohort and the test cohort both have been examined on the same MR scanner which might provide high consistency of image quality and scanner parameters between the training and test set facilitating generalization of the model to unseen specimens. However, data acquisition and subject recruitment for the two cohorts were conducted in the context of previous distinct and independent studies and at differing points in time. Sequence parameters have been updated in the context of the different main investigation endpoints of the respective studies. The test set therefore qualifies well as independent from the training set. Still, the constants of the scanner and environmental factors might be a source of elevated consistency between the two. Larger, more diverse multi-institutional datasets should therefore be the groundwork for future studies in this area.

Lastly, segmentation quality decreased in the more distal parts of the nerve(s) due to the fact that ambivalent pixels at the nerve boundaries and adjacent vessels have an increasing influence when the actual nerve diameter is smaller in comparison. This finding was currently assessed by visual inspection only. In future studies, this should be assessed in a more objective manner, along with an evaluation by extended robust measures, like balanced HD [40].

In conclusion, a U-net-based segmentation algorithm for the task of healthy sciatic nerve segmentation has been trained in this study, based on a comparably large dataset with high-quality ground truth labels. Overall, there were good performance and generalization capabilities on an independent test regarding standard segmentation performance measures and as well in terms of a segmentation-derived imaging feature of the segmented nerve. Future work will entail the extension of the training data to pathological nerve findings, more diverse and extensive datasets, and exhaustive evaluation of segmentation-derived imaging features for advanced artificial intelligence-supported disease characterization in peripheral neuropathies.

### Supplementary information

**Additional file 1: Supplementary Table S1:** Network architecture details. **Supplementary Table S2:** Normal distribution. **Supplementary Figure S1:** Training loss curves.

## Data Availability

Data supporting the results is not publicly available to avoid compromising the individual privacy of patients included in the clinical studies. To request data access, kindly ask the corresponding author via e-mail (Nedim Beste, nedim25@me.com).

## Abbreviations

2D: Two-dimensional
3D: Three-dimensional
CI: Confidence interval
DSC: Dice similarity coefficient
HD: Hausdorff distance
JI: Jaccard index
MR: Magnetic resonance
MRN: Magnetic resonance neurography
