# Supplementary material for: Automated peripheral nerve segmentation for MR-neurography
Source: Eur Radiol Exp. 2024 Aug 26;8:97. doi: 10.1186/s41747-024-00503-8 (PMC11347527; doi:10.1186/s41747-024-00503-8)
Supplement: Supplementary file 1 — Additional file 1: Supplementary Table S1: Network architecture details. Supplementary Table S2: Normal distribution. Supplementary Figure S1: Training loss curves. [file 41747_2024_503_MOESM1_ESM.pdf]

Automated peripheral nerve segmentation for MR-neurography

ELECTRONIC SUPPLEMENTARY MATERIAL

**Supplementary Table S1: Network architecture details**	**2D**	**3D**
**Downsampling strides**	[[2, 2], [2, 2], [2, 2], [2, 2], [2, 2], [2, 2], [2, 2]]	[[1, 2, 2], [1, 2, 2], [1, 2, 2], [2, 2, 2], [2, 2, 2], [1, 2, 2]]
**Convolution kernel strides**	[[3, 3], [3, 3], [3, 3], [3, 3], [3, 3], [3, 3], [3, 3], [3, 3]]	[[1, 3, 3], [1, 3, 3], [1, 3, 3], [3, 3, 3], [3, 3, 3], [3, 3, 3], [3, 3, 3]]

Architectural details for the segmentation networks. U-net shape with multiple resolution stages with two computational blocks per resolution stage in encoder and decoder. Each computational block contains convolution, instance normalization and leaky rectified linear unit (negative slope: 0.01) in this order. Downsampling is applied with strided convolutions and the following upsampling with transposed convolutions. The downsampling strides indicate the downsampling factors along the axes. For instance, a downsampling factor of 1 along all axes for the 3D model is expressed by [1, 1, 1]. Details regarding this notation can also be found in the Supplementary of the original nnUnet publication by Isensee et al. [20].

**Supplementary Table S2: Normal distribution**	**Model**	**Metric**	**W***	**p***
	**Training**			
		**DSC**	0.866	< 0.001
		**JI**	0.894	< 0.001
		**HD**	0.935	< 0.001
	**Test**			
		**DSC**	0.612	< 0.001
		**JI**	0.766	< 0.001
		**HD**	0.935	< 0.001
	**Intra-Rater**			
		**DSC**	0.919	< 0.001
		**JI**	0.923	< 0.001
*Shapiro Wilk test		**HD**	0.927	< 0.001

Supplementary Figure S1: Training loss curves

2D model

Fold 0

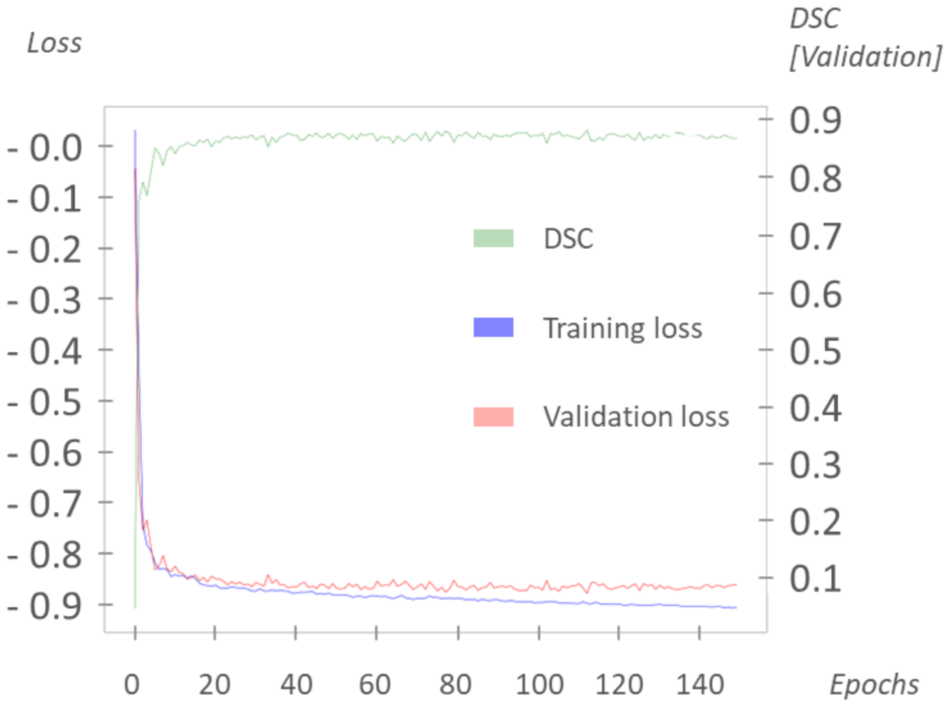

Fold 1

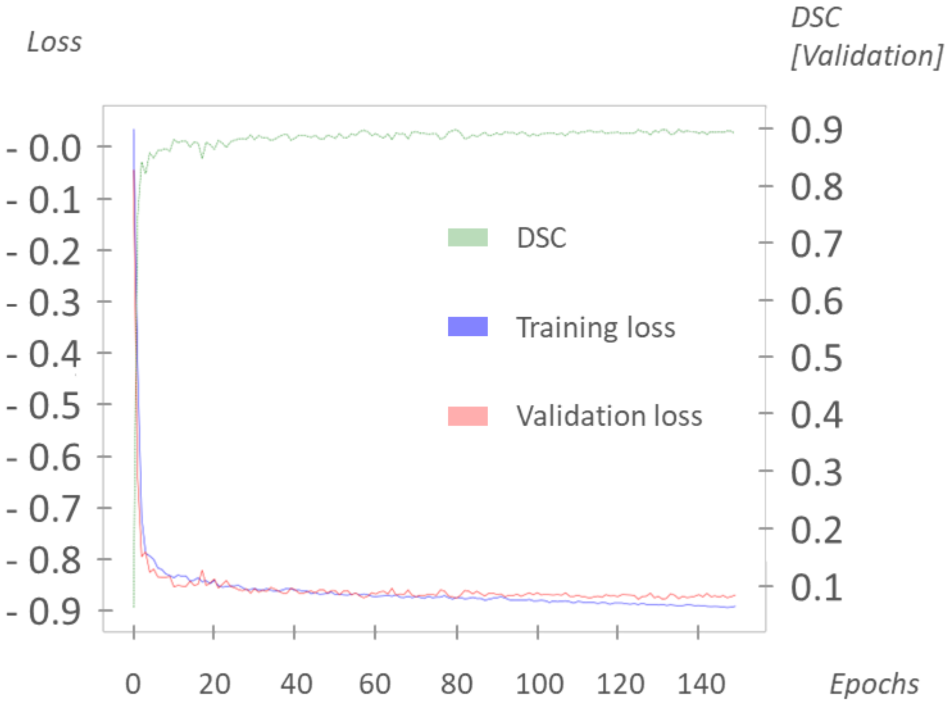

Fold 2

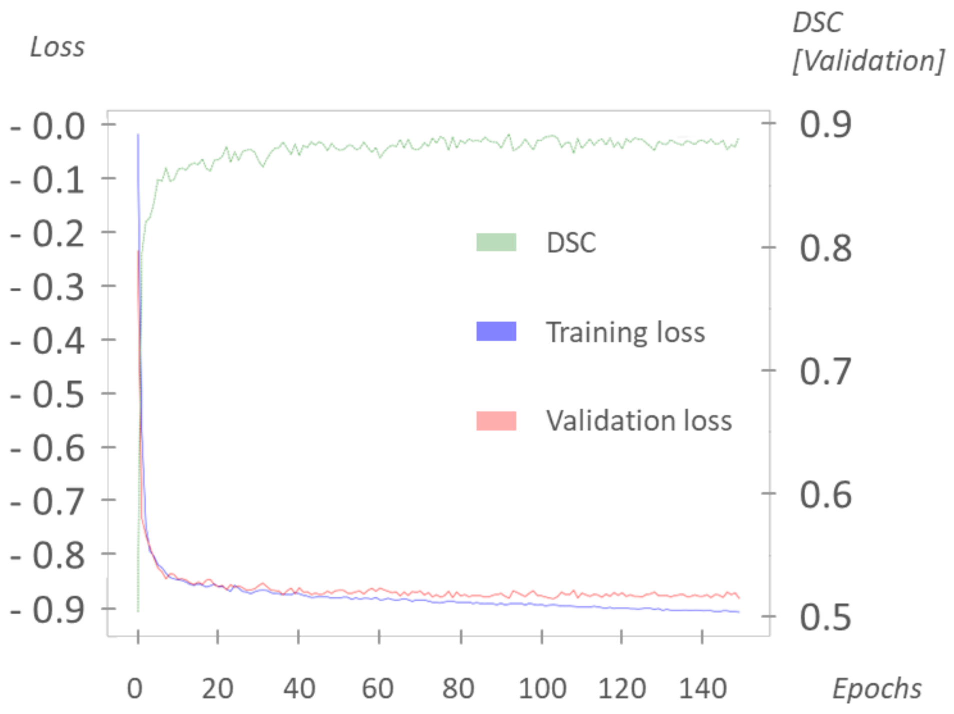
Loss
DSC [Validation]
- 0.0
- 0.1
- 0.2
- 0.3
- 0.4
- 0.5
- 0.6
- 0.7
- 0.8
- 0.9
0.9
0.8
0.7
0.6
0.5
DSC
Training loss
Validation loss
0
20
40
60
80
100
120
140
Epochs

Fold 3

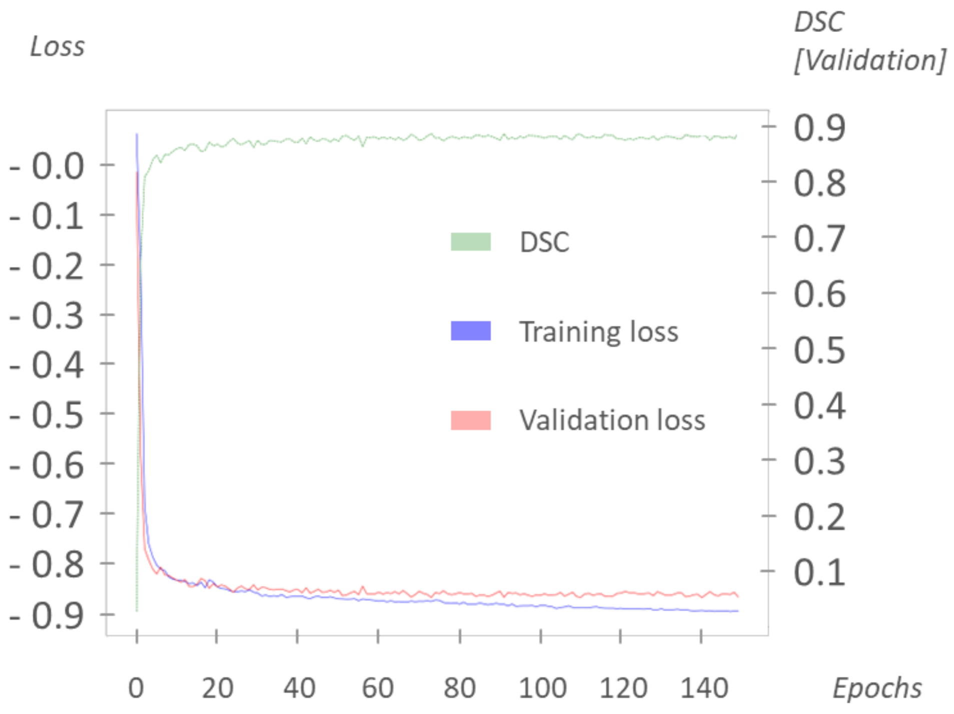
Loss
DSC [Validation]
- 0.0
- 0.1
- 0.2
- 0.3
- 0.4
- 0.5
- 0.6
- 0.7
- 0.8
- 0.9
0.9
0.8
0.7
0.6
0.5
0.4
0.3
0.2
0.1
DSC
Training loss
Validation loss
0
20
40
60
80
100
120
140
Epochs

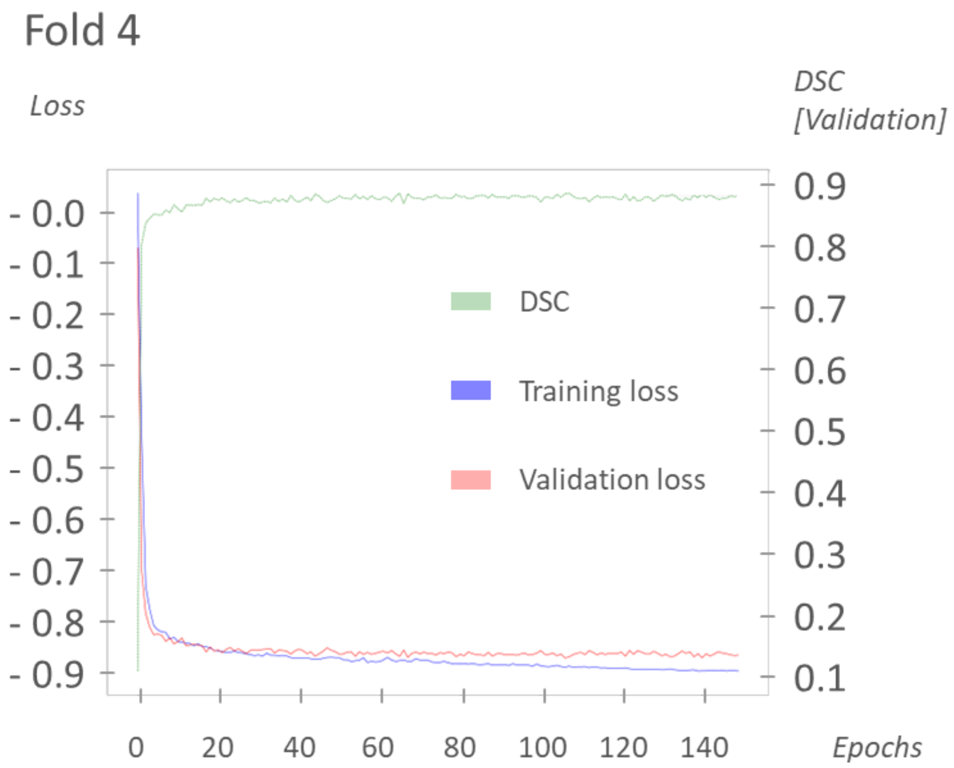

Fold 4
Loss
DSC [Validation]
- 0.0
- 0.1
- 0.2
- 0.3
- 0.4
- 0.5
- 0.6
- 0.7
- 0.8
- 0.9
0.9
0.8
0.7
0.6
0.5
0.4
0.3
0.2
0.1
DSC
Training loss
Validation loss
0
20
40
60
80
100
120
140
Epochs

3D model

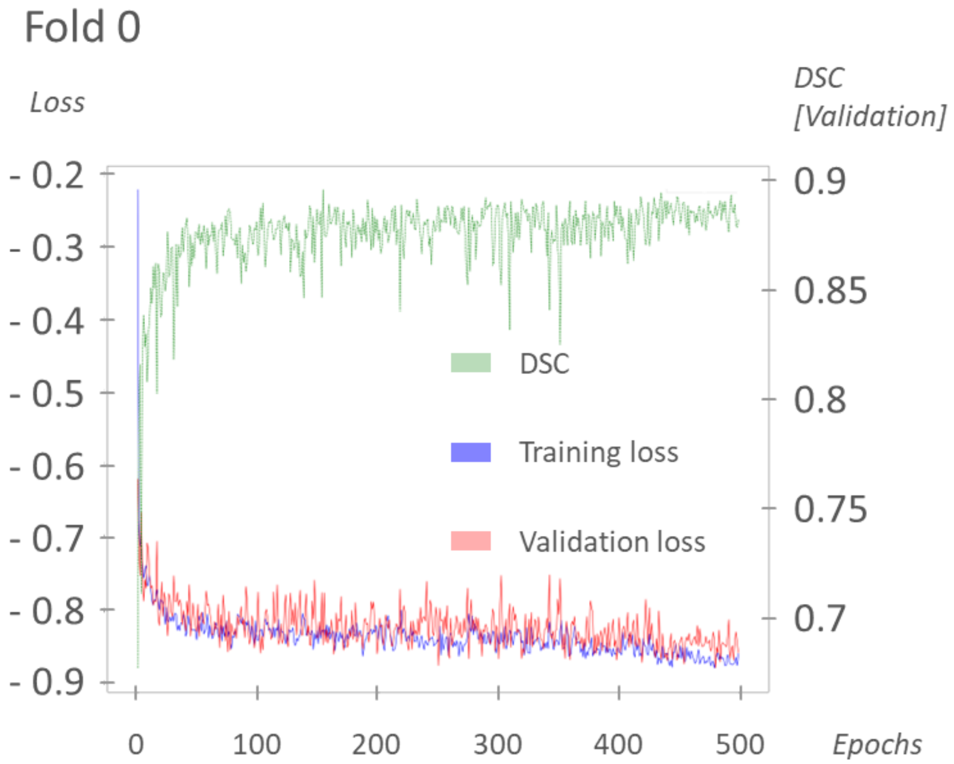

Fold 0
Loss
DSC [Validation]
- 0.2
- 0.3
- 0.4
- 0.5
- 0.6
- 0.7
- 0.8
- 0.9
0.9
0.85
0.8
0.75
0.7
DSC
Training loss
Validation loss
0
100
200
300
400
500
Epochs

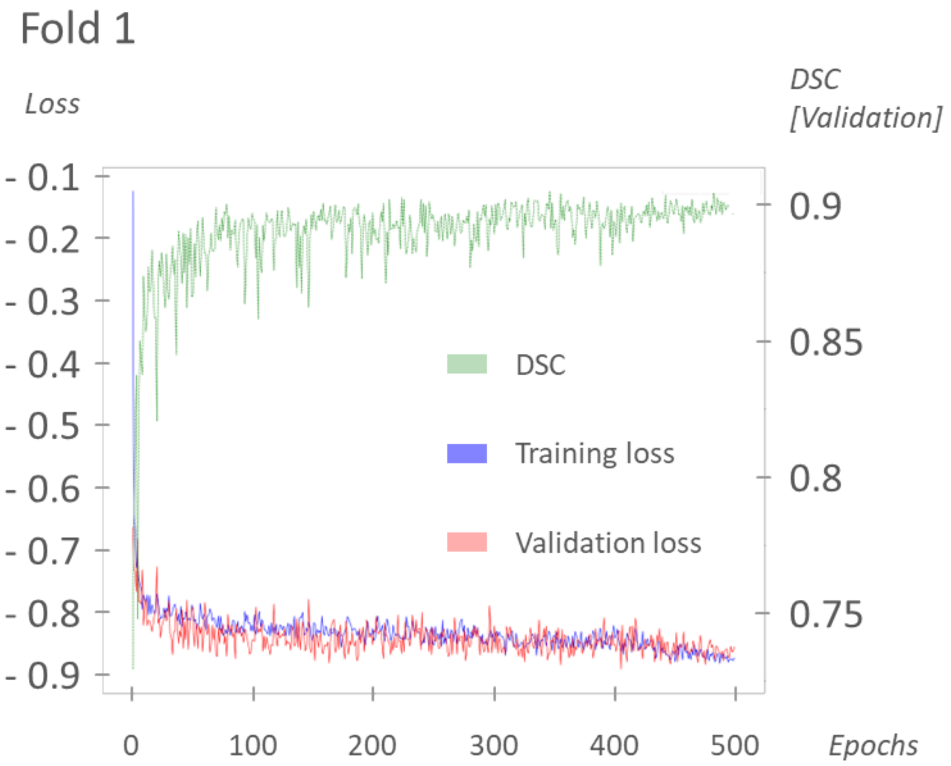
Fold 1
Loss
DSC [Validation]
- 0.1
- 0.2
- 0.3
- 0.4
- 0.5
- 0.6
- 0.7
- 0.8
- 0.9
0.9
0.85
0.8
0.75
DSC
Training loss
Validation loss
0
100
200
300
400
500
Epochs

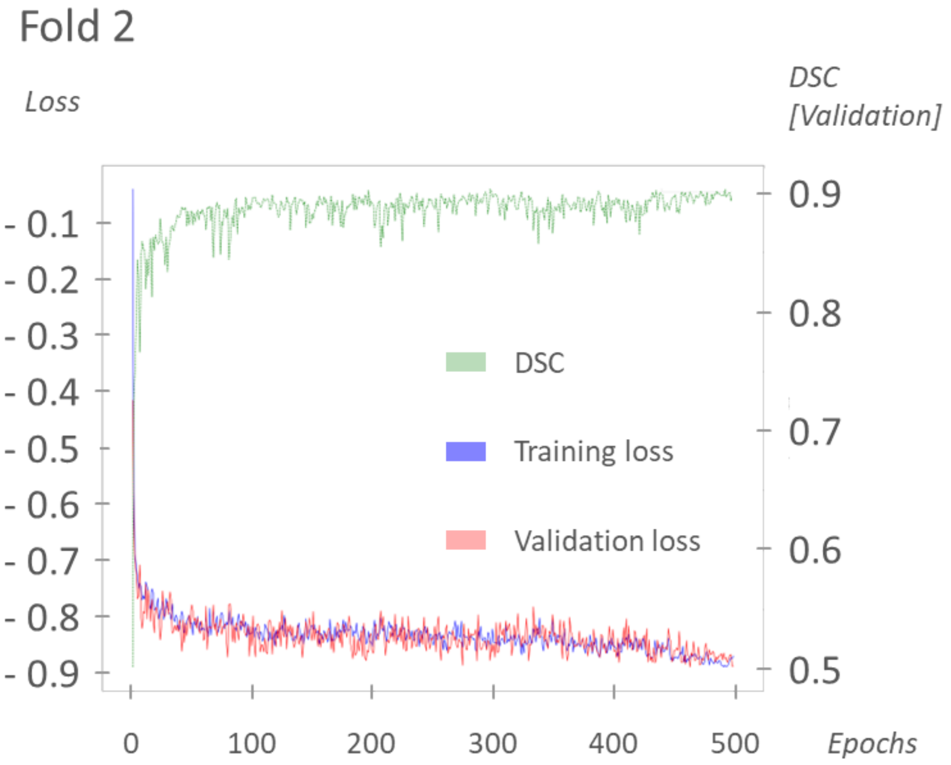
Fold 2
Loss
DSC [Validation]
- 0.1
- 0.2
- 0.3
- 0.4
- 0.5
- 0.6
- 0.7
- 0.8
- 0.9
0.9
0.8
0.7
0.6
0.5
DSC
Training loss
Validation loss
0
100
200
300
400
500
Epochs

Fold 3

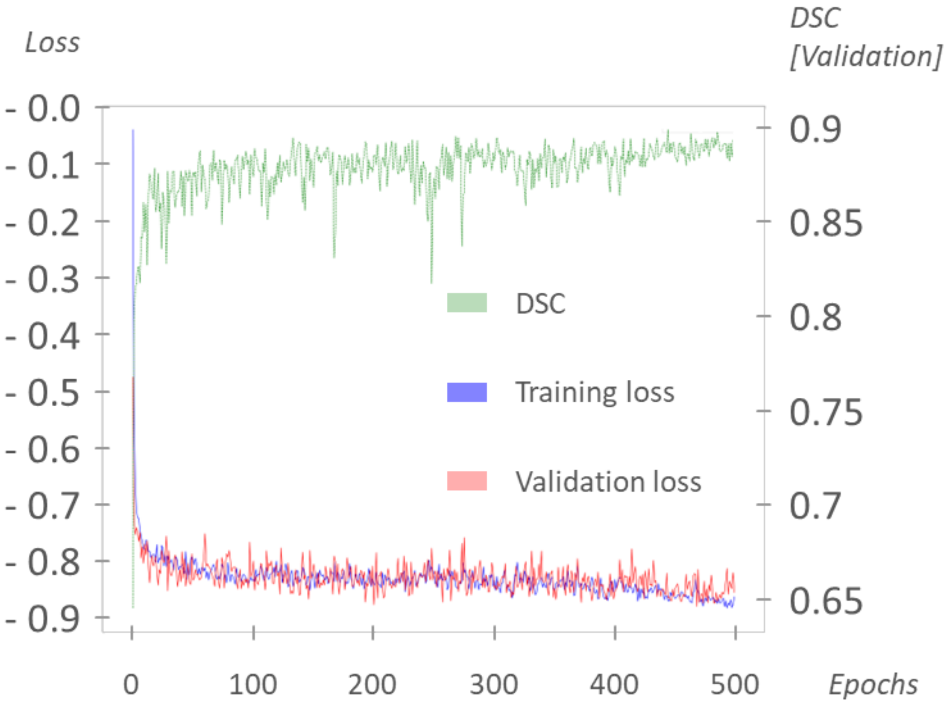
Loss
DSC [Validation]
- 0.0
- 0.1
- 0.2
- 0.3
- 0.4
- 0.5
- 0.6
- 0.7
- 0.8
- 0.9
0.9
0.85
0.8
0.75
0.7
0.65
DSC
Training loss
Validation loss
0
100
200
300
400
500
Epochs

Fold 4

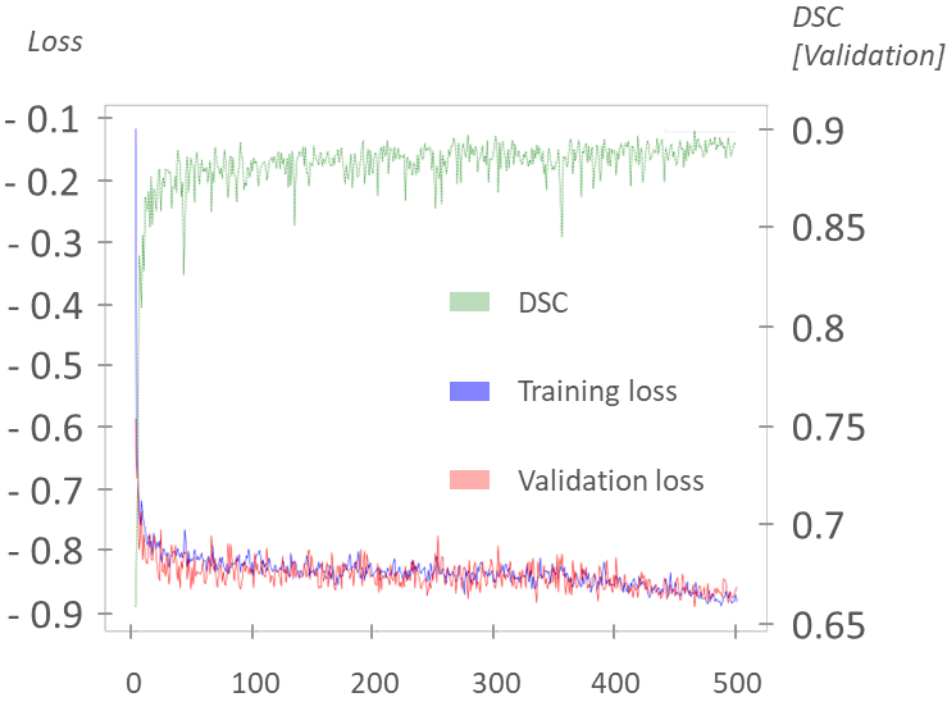
Loss
DSC [Validation]
- 0.1
- 0.2
- 0.3
- 0.4
- 0.5
- 0.6
- 0.7
- 0.8
- 0.9
0.9
0.85
0.8
0.75
0.7
0.65
DSC
Training loss
Validation loss
0
100
200
300
400
500
